# Recursive use of home ranges and seasonal shifts in foraging behavior by a generalist carnivore

**DOI:** 10.1002/ece3.9540

**Published:** 2022-11-24

**Authors:** Jordan L. Youngmann, Joseph W. Hinton, Nicholas W. Bakner, Michael J. Chamberlain, Gino J. D'Angelo

**Affiliations:** ^1^ Daniel B. Warnell School of Forestry and Natural Resources University of Georgia Athens Georgia USA; ^2^ Wolf Conservation Center South Salem New York USA

**Keywords:** *Canis latrans*, coyote, foraging, recursive behavior, resource selection, scavenging, space use

## Abstract

Coyotes (*Canis latrans*) colonized the southeastern United States over the last century as large predators, including the red wolf (*Canis rufus*) and eastern cougar (*Puma concolor*), were extirpated from the region. As a generalist carnivore, the coyote preys on white‐tailed deer (*Odocoileus virginianus*) and various smaller mammals, birds, and vegetation. While resource selection by coyotes has been well documented at the home‐range scale, little is known about their foraging behavior, which is an important factor in thoroughly understanding influences of coyotes on prey and sympatric carnivores. We assessed third‐order resource selection of coyotes at sites across Alabama, Georgia, and South Carolina during 2015–2016. Using GPS collars, we tracked 41 resident coyotes across four calendar seasons and identified suspected foraging areas using recursive analysis where individuals repeatedly returned to known locations. We found that resident coyotes selected for open landcover types throughout the year, while avoiding primary and secondary roads. Additionally, resident coyotes avoided forested landcover types while selecting for forest edges except from April to June when they foraged within interior forest away from edges. Previous studies have documented substantive predation rates on white‐tailed deer neonates by coyotes, and that fawn mortality may increase in forested landscapes away from forest edge. Our findings indicate that foraging coyotes may select forest cover types during spring where fawns are more vulnerable to predation. Additionally, there has been debate in the literature as to how coyotes obtain consistent levels of deer in their diets outside of fawning and fall hunting seasons. Our study indicates that use of road‐kill carcasses by coyotes was an unlikely explanation for the presence of deer in coyote diets throughout the year, as coyotes in our study were not observed using roads during foraging excursions.

## Graphical abstract

We assessed third‐order resource selection of coyotes across Alabama, Georgia, and South Carolina using recursive analysis to better understand nocturnal foraging behavior. We found that resident coyotes selected for open landcover types throughout the year, while avoiding primary and secondary roads and avoided forested landcover types while selecting for forest edges except from April to June when they foraged within interior forest away from edges. Our findings indicate that foraging coyotes may select forest cover types during spring where white‐tailed deer fawns are more vulnerable to predation and that use of road‐kill carcasses by coyotes was an unlikely explanation for the presence of deer in coyote diets throughout the year.

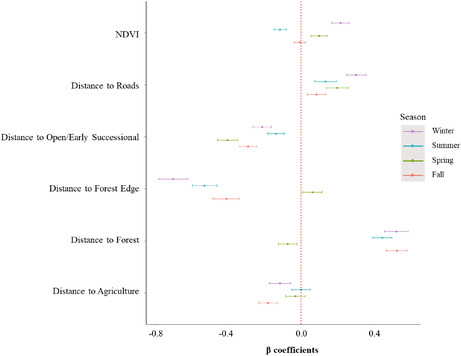

## 1 INTRODUCTION

Animals maintain home ranges through repeated visitations to areas in a systematic manner (Van Moorter et al., 2009, 2016). By maintaining a home range, animals minimize risks associated with navigating unfamiliar areas, while improving survival and fitness by revisiting areas that contain critical resources. Revisiting areas promotes disproportionate space use in home ranges, which minimizes extensive, wide‐ranging movements that would destabilize use of distinct core areas that shape the home range (McKeown et al., 2019; Merkle et al., 2014; Morin & Kelly, 2017; van Moorter et al., 2016). Depending on species, animals respond to spatio‐temporal dynamics of resources using various movement strategies that can range from nomadism (Teitelbaum & Mueller, 2019) to sedentatism (Sells & Mitchell, 2020). Understanding how recursive behaviors shape these movement strategies is an emerging area of ecological study that examines the repeated use of areas for resource acquisition (Berger‐Tal & Avgar, 2012; Berger‐Tal & Bar‐David, 2015; Ohashi & Thomson, 2005; Riotte‐Lambert et al., 2013).

Recursive behavior is routinely documented in a variety of species, occurring when animals remember where resources are located within a heterogenous landscape (Berger‐Tal & Avgar, 2012; Berger‐Tal & Bar‐David, 2015). Path recursion results from nonrandom movements in which animals monitor areas of foraging and repeatedly return to resource rich areas while ignoring resource poor areas (Berger‐Tal & Bar‐David, 2015). Although traditional resource selection analyses have made tremendous advancements in associating animal movements to available resources within their home ranges (Boyce et al., 2002; Boyce & McDonald, 1999; Manly et al., 2002), recursive analysis narrows the focus to areas of repeated use by animals. Therefore, recursive analysis can directly link a mechanism driving animal decision‐making (i.e., foraging, den site selection, travel corridors) and resource availability within home ranges.

As noted by Berger‐Tal and Bar‐David (2015), recursive movements are nearly universal in animals, but the phenomenon is given limited appreciation by researchers because there is little cross‐referencing among studies using different methodologies and nomenclature. Their synthesis reported parallels in studies on traplining behavior of pollinators, path recursion of migratory ungulates, and predator–prey interactions under the landscape of fear framework. Whether the study of recursive movements is conducted directly or through other parallel lines of research, it provides a powerful tool for us to understand behavioral and ecological processes. For example, McKeown et al. (2019) used recursive analysis to study movement behavior underlying the formation of red fox (*Vulpes vulpes*) home ranges in southeastern Sweden, whereas Buderman et al. (2018) incorporated recursive behaviors into resource selection functions (RSFs) to examine cougar (*Puma concolor*) ecology in the wildland–urban landscape of the Front Range of Colorado, USA.

In the United States, research on coyotes (*Canis latrans*) has increased significantly over the last several decades because of the species' recent range expansion (Hinton et al., 2019; Hody & Kays, 2018), role as the top canid predator in most regions (Gompper, 2002; Kilgo et al., 2010; Robinson et al., 2014), ability to live in urban areas (Breck et al., 2019; Gehrt et al., 2009; Lombardi et al., 2017), and hybridization with red wolves (*Canis rufus*; Bohling & Waits, 2015; Hinton et al., 2018; Nowak, 2002) and eastern wolves (*Canis lycaon*; Benson et al., 2012; Rutledge et al., 2012; Wilson et al., 2000). In the southeastern United States, average home range sizes reported for resident coyotes are relatively large (range = 5.2–85.0 km^2^; Chamberlain et al., 2021; Hickman et al., 2016; Hinton et al., 2015; Mastro et al., 2019; Stevenson et al., 2019; Ward et al., 2018) and consist of a diversity of land cover ranging from open anthropogenic (i.e., urban and agriculture) to dense vegetation cover (Hickman et al., 2016; Hinton et al., 2015; Stevenson et al., 2019; Ward et al., 2018). Recent studies of coyotes in the southeastern United States reported that coyotes tend to select early successional vegetation communities and open landcover types (Hinton et al., 2015; Stevenson et al., 2019) and primarily consume mammalian prey and fruit (Cherry et al., 2016; Hinton et al., 2017, 2021; Schrecengost et al., 2008; Ward et al., 2018). Ward et al. (2018) suggested that coyote home ranges were stable year‐round because of differential use of heterogenous resources (i.e., prey and land cover) and population dynamics of their preferred prey prevented coyotes from overexploiting limited resources within their home ranges. This observation aligns with some key prerequisites of recursive behavior in which foraging areas should show recovery after depletion, and that coyotes have spatio‐temporal memory to track the rate of resource recovery in their home ranges (Berger‐Tal & Bar‐David, 2015). Indeed, because coyotes actively defend their home ranges from conspecifics, presumably they have substantive knowledge of local conditions and spatio‐temporal patterns of revisitation to areas by coyotes (i.e., recursion behavior) underlies how coyotes form and maintain home ranges.

Despite previous work in the region on second‐ and third‐order resource selection by coyotes (Crimmins et al., 2012; Hickman et al., 2016; Hinton et al., 2015; Stevenson et al., 2019; Thornton et al., 2004), how coyote foraging is influenced by landscape characteristics remains poorly understood (Ward et al., 2018). A review of the breadth of coyote research across the eastern United States identified foraging ecology as an important area of future research (Mastro, 2011; Mastro et al., 2011). However, due to the densely forested landscapes of the eastern United States, it is difficult to assess coyote behavior without the use of remote VHF and GPS technologies and novel approaches to analyzing movement data may best capture important ecological behaviors. When correlating prey used by coyote packs with land cover characteristics of home ranges and mean monthly temperatures (e.g., season), Ward et al. (2018) found that vegetation density and season influenced which prey coyotes consumed. For example, coyote consumption of deer and rabbits was negatively correlated with vegetation density, whereas consumption of small mammals and fruit was positively correlated with vegetation density. However, when accounting for coyote consumption of fawns, Ward et al. (2018) reported that season was the most important factor and land cover provided little to no information. Given that our movement data were collected from the same study animals used by Ward et al. (2018), we believe our recursive analysis provides further insights into their findings and coyote foraging behavior in general.

Our objective was to assess the relationship between foraging behaviors of coyotes and landcover using a recursive analysis combined with RSFs in which we assumed that coyotes repeatedly visited areas to acquire resources. Ward et al. (2018) correlated prey use by coyotes with size of coyote home ranges as well as land cover types in their home ranges. The intent of the study was to blend traditional diet analysis with resource selection to make stronger inferences about factors influencing coyote diets. We build off Ward et al. (2018) by using recursive analysis to identify potential foraging areas within the coyote home ranges reported in their study and correlating land cover characteristics with these foraging areas rather than frequency of occurrence of prey types in pack diets. To accomplish this, we used nocturnal and crepuscular locations from coyotes studied by Ward et al. (2018), which consisted of 41 resident coyotes from 15 pack territories across Alabama, Georgia, and South Carolina, USA. In this region, coyotes were not typically active outside their loafing areas during the day (Hinton et al., 2015; Ward, 2017) and previous research has shown coyotes to be predominately nocturnal while foraging (Andelt & Andelt, 1981; Grinder & Krausman, 2001; Holzman et al., 1992), so we assumed most foraging occurred between dusk and dawn.

Due to the exploratory nature of our analysis and its use of previously published data, we had no genuine a priori hypotheses and worked under a predictive modeling framework rather than a hypothetico‐deductive one (Freedman, 1983; Tredennick et al., 2021). Indeed, our intent was not to conduct analyses on a familiar dataset and then report our goals as a priori hypotheses. Nevertheless, we believe our assessment is an important step for improving study designs and hypotheses investigating coyote ecology and their interactions with prey. We built global models for each season using land cover covariates known to be important to second‐ and third‐order coyote resource selection (Table 1). Coyotes in the southeastern United States have been shown to prefer open, early successional and agricultural landcover, while avoiding forested landcover (Chamberlain et al., 2000; Cherry et al., 2017; Hickman et al., 2016; Hinton et al., 2015; Holzman et al., 1992; Schrecengost et al., 2009; Stevenson et al., 2019; Ward et al., 2018). It is hypothesized that more open landscapes mirror the environments of coyotes in the western United States. We predicted that recursive movements associated with coyote foraging would be linked with these open landcover types, while coyotes would avoid forests for foraging. Several studies have shown that edge features are an important factor in home‐range selection (Hinton et al., 2015; Tigas et al., 2002; Webster, 2022) and it is generally understood that coyotes use landscape edges for hunting and navigation. We predicted that forest edges would be important for recursive movements during foraging bouts. Additionally, Chamberlain et al. (2021) found that coyotes used high‐density vegetation for both foraging as well as denning in the spring and we predicted that recursive behavior would be associated with increased vegetative density throughout the year. Finally, to assess if coyote consumption of deer occurred primarily through scavenging of roadkill, we used recursive analysis to correlate revisitation of areas proximate to roads during fall and winter when deer experience greater road mortality due to increased movements associated with their breeding season. Several diet studies have questioned if scavenging of roadkill is an important foraging strategy for coyotes and we predicted that we would see a negative correlation between recursive movements and proximity to roads.

**TABLE 1:** A selection of landcover covariates that potentially influence foraging behavior of coyotes in Alabama, Georgia, and South Carolina during 2015–2016

Covariate	Biological importance	References
Agriculture	Resident coyotes select for agriculture when choosing home ranges (second‐order selection) and use agriculture within their home ranges (third‐order selection).	Hinton et al. (2015) and Ward et al. (2018)
Open, early successional	Coyotes select for open, early successional habitat in their home ranges (third‐order selection)	Cherry et al. (2017), Hinton et al. (2015) and Stevenson et al. (2019)
Forest	Coyotes avoid forest cover in their home ranges (third‐order selection)	Chamberlain et al. (2000), Hickman et al. (2016), Hinton et al. (2015), Holzman et al. (1992) and Schrecengost et al. (2009)
Forest edge	Coyotes are known to forage along habitat edges and edge is an important factor in home‐range selection (second‐order selection)	Hinton et al. (2015) and Tigas et al. (2002)
NDVI	Coyotes may select high‐density vegetation for foraging	Chamberlain et al. (2021) and Ward et al. (2018)
Roads	Resident coyotes avoid roads in their home range selection (third‐order selection) but may use them for scavenging roadkill	Chamberlain and Leopold (1999), Cherry et al. (2016), Crimmins et al. (2012) and Schrecengost et al. (2008)

Abbreviation: NDVI, normalized difference vegetation index.

## 2 MATERIAL AND METHODS

### 2.1 Study area

**FIGURE 1:**
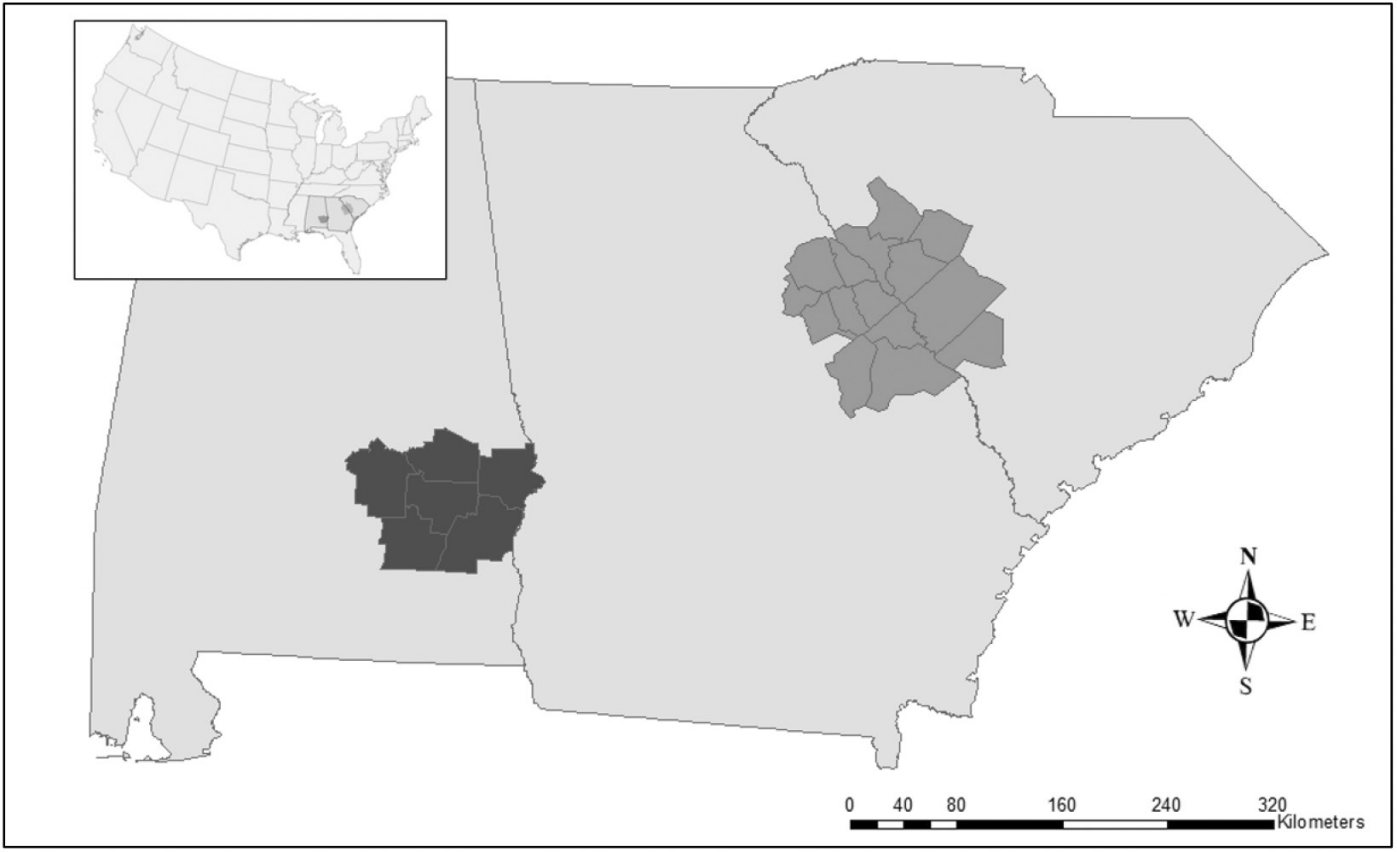
Map showing study sites in Alabama, Georgia, and South Carolina, United States used to assess recursive movements of coyotes in 2015–2016. The Alabama site is in dark gray on the Alabama‐Georgia line and the Savannah River Area (SRA) site is in light gray on the Georgia–South Carolina line.

We conducted research across approximately 16,200 km^2^ of public and private lands in Alabama, Georgia, and South Carolina, USA. Our study area was comprised of two distinct populations of coyotes: Alabama and the contiguous Georgia–South Carolina complex on both sides of the Savannah River (hereafter, SRA; Figure 1). The Alabama population was in the Southeastern Plains ecoregion along the southern border of the Piedmont ecoregion, whereas the SRA population occurred along the boundary between the Piedmont and the Southeastern Plains (Omernik, 1987).

The Southeastern Plains and Piedmont ecoregions were characterized by a mixture of upland hardwoods and pines (*Pinus* spp.), and bottomland hardwoods along drainage systems (Griffith, 2010). However, the Southeastern Plains included more loblolly pine forests (*P. taeda*) with the addition of oak–hickory–pine woodlands. Land use across both ecoregions was largely loblolly and shortleaf pine (*P. echinate*) plantations and agriculture dominated by cotton, corn, tobacco, soybeans, and peanuts, with the Southeastern Plains generally containing more land in agriculture. Our study area experienced mild, mid‐latitude humid subtropical climate with mean annual temperatures around 17°C (Griffith, 2010). Annual rainfall averaged 136 cm in the Southeastern Plains and 123 cm in the Piedmont (NOAA, 2018).

Available food for coyotes included deer, eastern wild turkeys (*Meleagris gallopavo*), rabbits (*Sylvilagus* spp.), squirrels (*Sciurus* spp.), eastern woodrats (*Neotoma floridana*), hispid cotton rats (*Sigmodon hispidus*), mice (*Peromyscus* spp.), shrews (*Blarina* spp., *Sorex* spp.), voles (*Microtus* spp.), wild pig (*Sus scrofa*), armadillos (*Dasypus novemcinctus*), opossums (*Didelphis virginiana*), insects, persimmons (*Diospyros virginiana*), blackberry (*Rubus* spp.), wild plums (*Prunus* spp.), pokeweed (*Phytolacca americana*), wild grape (*Vitis* spp.), muscadine (*Vitis rotundifolia*), and black cherry (*Prunus serotina*). Ward et al. (2018) documented diets of resident coyotes across our study sites and reported that of 1226 scat samples, 40.7% contained deer, 25.1% rabbits, 24.5% other small mammals, and 27.5% fruits such as persimmons, wild grape, muscadine, blackberry, dewberry, and pokeweed. Insects, armadillos, livestock, opossum, raccoon, birds, reptiles, human trash, and wild pigs were minor components of coyote diets.

### 2.2 Sampling design

We trapped coyotes during January–February 2015–2016 using offset MB‐550 foothold traps (Minnesota Trapline Products Inc.) and used catchpole, muzzle, and hobbles to restrain them. We fitted coyotes with mortality‐sensitive G2110E satellite collars (Iridium; Advanced Telemetry Systems) programmed to collect 4‐h interval fixes. Locations were transmitted every 3 days to an Advance Telemetry Systems website center through an Iridium Satellite system. Our research was conducted under approval of the University of Georgia Institutional Animal Care and Use Committee (A2014 08‐025‐R2) and we followed guidelines published by the American Society of Mammologists (Sikes & Gannon, 2011) and best management practices for trapping furbearers in the United States (White et al., 2021). For further information concerning field protocols and diet assessment see Ward et al. (2018).

### 2.3 Data analyses

To determine landscape‐level variables that influenced coyote foraging, we used a resource selection framework (Manly et al., 2002). First, we split our data across four seasons: spring (April–June), summer (July–September), fall (October–December), and winter (January–March). To account for seasonal changes in sunlight, we censored diurnal locations using the program “suncalc” in Program R 4.1.0 (R Core Team, 2020; Thieurmel & Elmarhraoui, 2019) by calculating dusk and dawn timestamps for each day and only including nocturnal and crepuscular locations between those times. We conducted our analyses on dusk to dawn locations to identify foraging locations for each individual coyote and excluded diurnal locations, which are likely associated with denning and loafing behaviors (Holzman et al., 1992).

**FIGURE 2:**
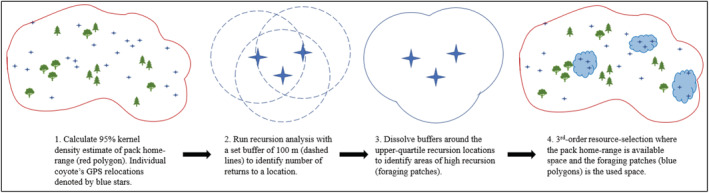
A workflow schematic detailing how third‐order resource selection is derived from individual coyote foraging movements using the program “recurse” in Program R 4.1.0 (Bracis et al., 2018).

We conducted recursion analyses using the program “recurse” in Program R 4.1.0 (Bracis et al., 2018; R Core Team, 2020) using a 100‐m buffer around each location to determine the number of times an individual returned to an area (hereafter, recursions) by season. We chose this buffer radius as a conservative estimate of the forage area size likely associated with foraging behaviors. Webster (2022) recently calculated first‐passage time (FPT) for a dataset that included the same individual coyotes as those in our study. They reported an average FPT radius around 1500‐m using the same 4‐h interval GPS collars. However, Bracis et al. (2018) warns against using a recursive buffer that results in many overlapping circles and suggests that behaviors such as foraging may require smaller buffers to best capture recursive movements. As a test of our buffer size, we ran recursive analyses at 100‐m, 250‐m, 500‐m, 750‐m, and 1000‐m radius intervals. We observed that buffers 250‐m and 500‐m created recursion polygons that closely mirrored the 50% KUD pack home range, while buffers 750‐m and 1000‐m closely mirrored, or exceeded, the 95% pack home range. Chamberlain et al. (2021) used first‐passage time analysis to quantify behavioral states of resident and transient coyotes. They noted that the largest mean variance for all movement paths in their study was 164.7 m and we felt comfortable using the aforementioned 100‐m buffer size in our analyses. Additionally, fix accuracy for our collars was 20 meters, making our buffer size appropriate to account for GPS error. Finally, for subsequent RSF analysis, we combined areas of high recursion to create foraging patches that were typically larger than our 100‐m buffer, making our choice of buffer size simply a tool for identifying areas of high return (Figure 2).

To determine used and random areas for RSFs, we first combined locations from all individuals within a pack to calculate seasonal 95% fixed kernel density estimates from utilization distributions for coyote pack home ranges using the *kernelUD* function in package “adehabitatHR” under the ad hoc smoothing parameter in Program R 4.1.0 (Calenge, 2006; R Core Team, 2020; Figure 2). We then selected pack GPS locations by season with total associated revisits calculated by “recurse” within the upper‐quartile range of recursions to represent points of high foraging use across pack home ranges. We buffered these locations by 100 m in R using the “raster” package with the dissolve function to create used areas and extracted all GPS locations from within these areas as used locations (Figure 2). To obtain summary statistics for recursion areas, we summed the total number of recursions per buffered location and used the package “recurse” in Program R 4.1.0 to estimate how much time (h) each coyote spent within that buffered area, and the time since the last previous visit to that location. Time inside a buffered area is calculated in the package “recurse” using linear interpolation between adjacent locations, which can allow for estimates smaller than the GPS fix intervals (Bracis et al., 2018). Once foraging areas were identified, we used the *spsample* function to sample all cells across the 95% kernel utilization distribution (KUD) pack home range to represent resource availability (Pebesma & Bivand, 2005; R Core Team, 2020). Systematic sampling of availability has been found to most accurately assess resource availability and produced satisfactory model convergence (Benson, 2013).

Our landcover covariates were chosen based on their known importance in second‐ and third‐order selection of cover types by coyotes in the southeastern United States (Table 1). To determine the influence of various landscape characteristics and landcover types on recursive movements, we obtained spatial data on vegetation density, distance to primary and secondary roads, and distance to landcover types. We identified and grouped primary and secondary roads using 2019 USGS Tiger/Line data (Topologically Integrated Geographic Encoding and Referencing). We used the normalized difference vegetation index (NDVI; Pettorelli et al., 2005) to estimate vegetation density and the 2016 National Land Cover Dataset (NLCD; Homer et al., 2015) to assess landcover types. We reclassified NLCD data to combine deciduous, evergreen, and mixed forests into a forest class; shrub/scrub and herbaceous into an open class; and hay/pasture and cultivated crops into an agriculture class. We also included distance to forest edge as a covariate because our study sites were predominately forested, and forest edge comprised most obvious ecotones within pack home ranges. We used the Euclidean Distance function in ArcGIS to calculate distance to roads and each landcover type for every 30‐m × 30‐m pixel across our study area. We extracted landscape‐level covariates across used and random locations to develop RSFs.

To assess the explanatory power of landscape covariates on foraging behavior, we conducted generalized linear mixed effect models in which use was a binary (1 = use, 0 = random) response variable and landscape covariates were explanatory variables using the package “lme4” in Program R 4.1.0 (Douglas et al., 2015). We tested our covariates by season for collinearity using the Spearman's correlation test, but all combinations retained a value of *r* < .6. Additionally, we included individuals nested within packs as a random intercept to address variation among individuals and packs. Due to the exploratory nature of our study, we chose to not employ a model selection methodology but, instead, used a global model from each season using the covariates selected for their known importance in eastern coyote ecology. Finally, we employed a modified version of Boyce et al. (2002) k‐fold cross‐validation method described in Roberts et al. (2017) to assess model performance. We blocked our data by pack so that each fold contained spatially independent individuals and averaged Spearman's rank correlation tests for each fold to assess model performance.

## 3 RESULTS

In our recursive analysis, we used 41 resident coyotes monitored via GPS collars during 2015–2016, 23 in the Alabama region and 18 in the SRA region. Across all seasons, recursions in our used points ranged from 1 (never returning to the location) to 37 (8.1 ± 5.9, mean ± SE, Table S1). Recursions were highest during spring (9.8 ± 6.5) and lowest during fall (7.3 ± 5.4). Time since return and time spent within a foraging area had positively skewed distributions, so we present median and interquartile ranges (IQR). Coyotes returned to foraging areas at a median interval of every 7.5 days (IQR = 4.1–16.0 days, Table S1). By season, coyotes returned to foraging areas at the shortest interval during spring (median = 5.9, IQR = 3.2–13.2 days) and the longest interval during summer (median = 8.6, IQR = 5.0–17.5 days). Individuals stayed within a recursive area for a median length of 4.5 h (IQR = 2.6–7.1 h, Table S1). Across seasons, coyotes remained within recursive areas least during summer (median = 3.8, IQR = 2.3–5.4 h) and most during spring (median = 6.3, IQR = 3.9–8.9 h).

### 3.1 Resource selection by season

**FIGURE 3:**
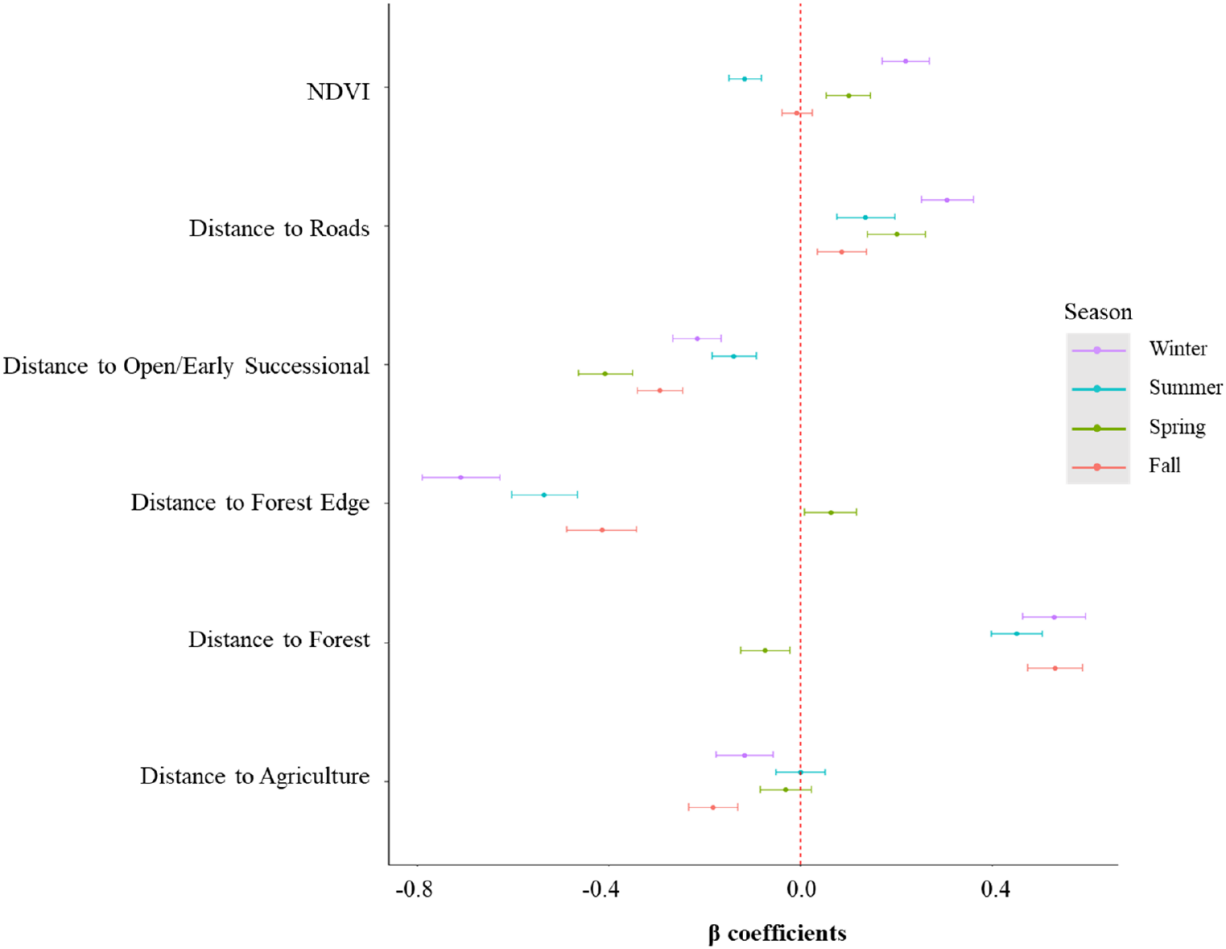
Parameter estimates with 95% confidence intervals for third‐order resource selection functions across four seasons for radio‐collared coyotes in Alabama, Georgia, and South Carolina during 2015–2016. For all distance‐based variables, negative values infer selection. For the normalized difference vegetation index (NDVI), positive values infer selection.

By season, we assessed third‐order resource selection of 41 individual coyotes across 15 packs within purported forage patches. Coyotes selected for agriculture during the fall and winter while neither selecting nor avoiding it during the spring and summer (Table 2, Figure 3). Coyotes avoided forest while selecting for forest edge in all seasons except spring, where they selected for forest while avoiding forest edge. Coyotes selected for open, early successional landcover while avoiding roads for every season. Finally, coyotes selected for less dense vegetation in the summer, higher vegetative density during the spring and winter, and showed no selection preference for vegetative density in the fall. Our models performed relatively well with average Spearman's rank correlation values ranging from 0.62 to 0.87 for each season (Table 2).

**TABLE 2:** Parameter estimates from top generalized linear mixed models for third‐order resource selection functions for radio‐collared coyotes in Alabama, Georgia, and South Carolina during 2015–2016.

Season	Model variables	*β*	SE	95% CI	*z*	*p*	*ρ*
Spring	Distance to forest	−.074	0.026	−0.125, −0.023	−2.840	.005	.62
	Distance to agriculture	−.031	0.027	−0.084, 0.022	−1.155	.248	–
	Distance to open/early	−.407	0.029	−0.463, −0.352	−14.291	<.001	–
	Distance to roads	.201	0.031	0.141, 0.261	6.526	<.001	–
	NDVI	.101	0.024	0.054, 0.147	4.267	<.001	–
	Distance to forest edge	.063	0.028	0.009, 0.117	2.275	.023	–
Summer	Distance to forest	.452	0.028	0.398, 0.506	16.419	<.001	.65
	Distance to agriculture	.000	0.026	−0.051, 0.051	−0.013	.990	–
	Distance to open/early	−.139	0.024	−0.185, −0.092	−5.865	<.001	–
	Distance to roads	.136	0.031	0.076, 0.196	4.457	<.001	–
	NDVI	−.116	0.017	−0.150, −0.082	−6.660	<.001	–
	Distance to forest edge	−.534	0.035	−0.603, −0.466	−15.264	<.001	–
Fall	Distance to forest	.532	0.029	0.474, 0.590	18.080	<.001	.69
	Distance to agriculture	−.183	0.026	−0.234, −0.132	−7.062	<.001	–
	Distance to open/early	−.293	0.024	−0.340, −0.245	−12.097	<.001	–
	Distance to roads	.087	0.026	0.035, 0.138	3.306	.001	–
	NDVI	−.008	0.016	−0.040, 0.025	−0.459	.646	–
	Distance to forest edge	−.415	0.037	−0.488, −0.342	−11.164	<.001	–
Winter	Distance to forest	.529	0.034	0.463, 0.595	15.705	<.001	.87
	Distance to agriculture	−.117	0.030	−0.176, −0.057	−3.837	<.001	–
	Distance to open/early	−.216	0.025	−0.266, −0.167	−8.561	<.001	–
	Distance to roads	.307	0.028	0.252, 0.362	10.921	<.001	–
	NDVI	.219	0.025	0.170, 0.268	8.750	<.001	–
	Distance to forest edge	−.709	0.041	−0.790, −0.629	−17.198	<.001	–

*Note*: Shown are coefficient estimates (*β*), standard error (SE), 95% confidence intervals (CI), *z*‐scores, and *p*‐values. Also included is the average Spearman's rank correlation coefficient (*ρ*) for each seasonal global model.

Abbreviation: NDVI, normalized difference vegetation index.

## 4 DISCUSSION

We used recursive analysis to identify areas where coyotes were presumed to be foraging and found that coyotes exhibited disproportional use of their home ranges because of their preferential use of land cover types. By censoring movement data to nighttime activity and using sites of recursive behavior as our selected foraging areas, we sought to identify foraging excursions among resident coyotes. A disadvantage of our methodology is that behaviors associated with recursive movements can be difficult to interpret. We recognize that our results may capture additional behaviors such as traveling, denning, and resting. However, our characterization of recursive movements during nocturnal and crepuscular hours as foraging behavior is aligned with studies that reported increased foraging activity of coyotes during these hours (Andelt & Andelt, 1981; Grinder & Krausman, 2001; Holzman et al., 1992). We found that coyotes used recursive areas at intervals ranging from approximately 4 days to more than 2 weeks. Coyotes foraged within these areas for a median length of approximately 4 h, the time interval that our GPS collars returned location fixes. Stated differently, our findings suggest that coyotes often moved and foraged continuously throughout their home range, rather than remaining within an area >4 h. We believe this pattern may reflect a movement strategy by coyotes to minimize time spent in areas, so as to reduce their exposure to mortality risks (predominately anthropogenic across our study area) while allowing prey to sufficiently recover between foraging bouts under a landscape of fear framework (Brown et al., 1999; Laundré et al., 2001).

Our general findings are consistent with other studies of coyote habitat selection conducted throughout the southern United States, in which coyotes typically select for open, early successional land cover (Cherry et al., 2016; Hinton et al., 2015; Holzman et al., 1992; Stevenson et al., 2019). Coyotes revisited open land cover across all seasons while exhibiting seasonal variation in revisitation to agriculture and forests. We found that coyotes selected for agriculture during revisitations only during fall and winter. Hinton et al. (2015) reported that coyotes used agricultural fields during the spring and summer as daytime loafing areas when planted crops grew high enough to provide cover. However, our findings indicate that agricultural fields were not used for foraging during this time. Cherry et al. (2016) found increased consumption of crops in coyote diets during fall and when fruit was locally unavailable. Coyotes may forage in agricultural fields during fall and winter to supplement their diet with residues from crops found in harvested fields, which could further explain the selection of agricultural cover by coyotes during winter, when plant consumption was at its lowest across packs presumably due to lack of native vegetation (Ward et al., 2018). However, it is also likely that coyotes exploit agricultural‐forest edges to hunt prey and consumption of crops is either acquired passively through consumption of prey or through scrounging in preferred foraging areas.

Coyotes selected areas of increased vegetation density during spring and winter revisitations, while avoiding areas of high vegetative density during the summer. Selection for areas with dense vegetation during spring was also observed in an independent study of coyote movements in north‐central Georgia, approximately 125 and 275 km from our SRA and Alabama study areas, respectively (Chamberlain et al., 2021). Using a behavioral state model, Chamberlain et al. (2021) reported that resident coyotes selected areas with dense vegetation density when resting or foraging during the pup‐rearing season. Therefore, it was not surprising to us that coyotes exhibited selection for dense vegetation during spring in our study for two reasons. First, coyotes whelp and care for small pups during March through June, and likely relied on vegetation cover for concealing their pups (Andelt, 1985; Chamberlain et al., 2021; Harrison & Gilbert, 1985; Mastro, 2011; Mastro et al., 2011; Way et al., 2001). Second, coyotes prey on deer and likely select cover types during spring that increase their ability to find fawns (Chamberlain et al., 2021; Ward et al., 2018). Deer are known to place their fawns in areas where fawns are concealed by vegetation (Gulsby et al., 2018; Shuman et al., 2018). As coyote pups and deer fawns increase in age and body size, their mobility improves, and they become more independent of their parents while relying less on vegetation concealment for security. Coyotes likely respond to this improved mobility by reorienting their movements to forest edges and adjacent cover types for the remainder of the year.

We documented selection for forested cover by coyotes and avoidance of forest edge during spring revisitations, suggesting that coyotes foraged under forest cover during this season. Our findings suggest that coyotes shift their selection towards interior forest during spring, possibly as both pup‐rearing and foraging strategies. Gulsby et al. (2017) reported a positive relationship between coyote depredation of fawns and mean patch size of forest, although they stated that reasons for the relationship between fawn survival and edge habitat, landscape heterogeneity, and forest patch size remain unclear. We believe selection by coyotes for interior areas of forest during their pup rearing season could be a mechanism by which fawns become more susceptible to predation in forested landscapes during peak fawning in the southeastern United States (April–June).

Finally, we documented avoidance of roads by resident coyotes throughout the year during revisitation bouts. Avoidance of roads by resident coyotes was also observed by Hinton et al. (2015) when they accounted for space use status (i.e., resident vs. transient) of study animals. Considering we only observed movements of resident coyotes, we believe these findings support Hinton et al.'s (2015) conclusions that transient coyotes (i.e., dispersing animals) rely more on road networks as movement corridors than do residents. Regardless, year‐round avoidance of roads by resident coyotes provides some insight into how coyotes may acquire deer in their diets throughout the year. For example, scavenging roadkill carcasses has often been implied as a means for coyotes to acquire adult deer (Chamberlain & Leopold, 1999; Crimmins et al., 2012; Schrecengost et al., 2008). Considering deer‐vehicle collisions typically peak during the fall and winter (Steiner et al., 2014; Stickles et al., 2015), we expected coyote foraging to shift towards roads during fall and winter to capitalize on the availability of roadkill carcasses. However, road avoidance by coyotes was similar from spring to fall before increasing during winter, when roadkill carcasses would have been better preserved along roads. Furthermore, roads are a primary source of mortality for many wildlife species and there is no shortage of animal carcasses for coyotes to scavenge along roadsides. If scavenging was an important foraging strategy for coyotes to acquire food, we would have expected to see recursive movements along roadways. Instead, we observed avoidance of roads by coyotes, which supports Hinton et al.'s (2017) suggestion that mortality risks for coyotes when traveling along roadways outweighed benefits of feeding on roadkill carcasses. Finally, Ward et al. (2018) found that consumption of deer by our study animals was associated with smaller home range sizes, indicating that coyote space use was negatively correlated with consumption of deer. It is unlikely that reduced space use would improve the ability of coyotes to locate carcasses spread intermittently along roadways. Nevertheless, deer were consumed year‐round by coyotes and the negative correlation between space use and consumption of deer implies that, regardless of season, coyotes selected land cover types where deer were most vulnerable to direct predation. During the spring, this would be land cover types where fawns were present, and during the rest of the year, areas where juvenile or adult deer were more accessible.

An important limitation to our study is the inability to distinguish individual behaviors using our movement dataset. Although we censored our data to most accurately capture known periods of coyote foraging (Andelt & Andelt, 1981; Grinder & Krausman, 2001; Holzman et al., 1992), it is likely that our recursive analysis captures some additional behaviors such as denning, territory marking, use of cover, and traveling. Previous work on movement recursions has often relied on ground truthing areas of repeated use to corroborate den or kill sites, water holes, and other areas of interest (Berger‐Tal & Bar‐David, 2015; Buderman et al., 2018; McKeown et al., 2019). Berger‐Tal and Bar‐David (2015) recognized the need for experimental validation of theoretical models because of the correlative association between recursion and behavior. Due to the plastic and mobile nature of our study species, future work is necessary to further tease out behavioral states and associated recursive movements. This may include behavioral‐state modeling with finer scale data to focus recursive analysis on empirically determined behaviors as well as fourth‐order resource selection that correlates diet consumed with the landscape covariates associated with recursive foraging patches, similar to Ward et al. (2018). Additionally, little work has been done to ground truth denning sites of coyotes in the eastern United States (Mastro, 2011; Mastro et al., 2011), which would help to better understand denning behavior of coyote packs during the spring and how they forage and use resources during that time. Here, our work attempts to provide a methodology using GPS data to explore recursive behavior in coyotes and seeks to inform hypotheses for future work on coyote ecology in the eastern United States.

## 5 CONCLUSIONS

Our research demonstrates that recursive analysis can be used to empirically explore foraging behavior of coyotes and can be used to identify movement corridors and suitable habitat for other species. Recursive movements and the rate of revisitations to foraging areas are necessary for coyotes to stabilize their movements and form home ranges. Furthermore, bounded space use and recursive movements to foraging areas demonstrate that resident coyotes exploit food resources within the spatial scale of their home ranges. Additionally, changes in selection across seasons may reveal a mechanism by which coyotes exhibit prey‐switching in response to changing availability of food resources. Although assessing the cognitive abilities of coyotes was beyond the scope of this study, it is obvious that memory‐based foraging and limits to vagility are responsible for how coyotes interact with landscape heterogeneity and resource dispersion as well as limiting the size of their home ranges.

## AUTHOR CONTRIBUTIONS

**Jordan L. Youngmann:** Conceptualization (equal); data curation (equal); formal analysis (lead); investigation (equal); methodology (equal); visualization (lead); writing – original draft (lead); writing – review and editing (equal). **Joseph W. Hinton:** Conceptualization (equal); data curation (equal); formal analysis (supporting); investigation (equal); methodology (equal); project administration (equal); supervision (equal); writing – original draft (supporting); writing – review and editing (equal). **Nicholas W. Bakner:** Conceptualization (equal); formal analysis (supporting); investigation (equal); methodology (equal); writing – original draft (supporting); writing – review and editing (equal). **Michael J. Chamberlain:** Conceptualization (equal); funding acquisition (lead); project administration (equal); resources (lead); supervision (equal); writing – original draft (supporting); writing – review and editing (equal). **Gino J. D'Angelo:** Conceptualization (equal); supervision (equal); writing – original draft (supporting); writing – review and editing (equal).

## CONFLICT OF INTEREST

The authors have no competing interests to declare.

## Supporting information


Table S1

## Data Availability

The data files for all movement analyses are available upon request or can be accessed on Dryad (https://doi.org/10.5061/dryad.z8w9ghxgh).
