# Supplementary material for: Recursive use of home ranges and seasonal shifts in foraging behavior by a generalist carnivore
Source: Ecol Evol. 2022 Nov 24;12(11):e9540. doi: 10.1002/ece3.9540 (PMC9685673; doi:10.1002/ece3.9540)
Supplement: Supplementary file 1 — Table S1 [file ECE3-12-e9540-s001.docx]

Supplementary Table 1. Summary statistics of recursions, time since last visit, time spent within recursion site, and frequency of occurrence of diet items found in pack scat radio-collared coyotes in Alabama, Georgia, and South Carolina during 2015-2016. Recursion Q3 cutoff is the third-quartile limit used to select high recursion GPS points to create forage patches. Summary statistics are otherwise derived from all GPS locations extracted from within the recursion patches.

|  |  | Recursion |  | Recursions | |  | Time Since | | |  | Time Inside | | |  | Average frequency of occurrence of diet | | | |
| --- | --- | --- | --- | --- | --- | --- | --- | --- | --- | --- | --- | --- | --- | --- | --- | --- | --- | --- |
| Season | Pack | Q3 Cutoff |  | Mean | SD |  | Q1 | Median | Q3 |  | Q1 | Median | Q3 |  | Deer^a^ | SM^b^ | Rabbit | Plants |
| Fall | Burks Mountain | 3 |  | 5.6 | 3.5 |  | 6.4 | 8.7 | 16.7 |  | 1.4 | 2.2 | 4.7 |  | NA | NA | NA | NA |
| Fall | Childs | 4 |  | 12.2 | 7.5 |  | 2.5 | 5.3 | 23.8 |  | 4.7 | 6.5 | 8.9 |  | 0.0 | 0.0 | 0.7 | 1.0 |
| Fall | Clayton Creek | 3 |  | 4.9 | 2.6 |  | 12.9 | 30.5 | 89.1 |  | 1.4 | 2.4 | 3.9 |  | 0.5 | 0.1 | 0.2 | 0.5 |
| Fall | Dunn | 5 |  | 7.0 | 3.2 |  | 4.4 | 6.6 | 9.5 |  | 2.7 | 3.6 | 4.6 |  | NA | NA | NA | NA |
| Fall | Gaston | 1 |  | 2.7 | 1.2 |  | 2.9 | 3.8 | 13.4 |  | 1.0 | 1.6 | 2.8 |  | NA | NA | NA | NA |
| Fall | Grant | 3 |  | 4.2 | 2.3 |  | 4.2 | 7.7 | 13.8 |  | 2.2 | 3.4 | 5.3 |  | NA | NA | NA | NA |
| Fall | Hwy 82 | 3 |  | 3.8 | 1.5 |  | 13.4 | 16.7 | 31.2 |  | 1.1 | 1.7 | 3.5 |  | 0.4 | 0.1 | 0.3 | 0.7 |
| Fall | Joyes | 1 |  | 4.3 | 3.9 |  | 3.0 | 4.7 | 8.9 |  | 1.6 | 3.0 | 5.3 |  | NA | NA | NA | NA |
| Fall | Kitchens | 6 |  | 6.3 | 1.8 |  | 4.1 | 8.0 | 12.1 |  | 2.9 | 3.7 | 4.5 |  | 0.3 | 0.1 | 0.5 | 0.4 |
| Fall | Mt. Calvary | 3 |  | 5.5 | 3.7 |  | 6.1 | 11.2 | 18.8 |  | 1.5 | 2.8 | 4.7 |  | 1.0 | 0.0 | 0.0 | 0.0 |
| Fall | Parker Hill | 4 |  | 6.6 | 3.4 |  | 3.2 | 5.3 | 8.6 |  | 3.5 | 4.5 | 5.6 |  | NA | NA | NA | NA |
| Fall | Pines Grove | 6 |  | 8.2 | 3.5 |  | 4.1 | 6.1 | 10.4 |  | 3.2 | 4.5 | 6.4 |  | 0.5 | 0.0 | 0.5 | 0.0 |
| Fall | Saluda | 4 |  | 6.6 | 3.3 |  | 6.2 | 9.5 | 13.3 |  | 1.6 | 2.2 | 3.7 |  | 0.4 | 0.1 | 0.3 | 0.3 |
| Fall | Soap Creek | 7 |  | 8.6 | 3.4 |  | 5.3 | 7.2 | 10.4 |  | 3.5 | 4.5 | 5.4 |  | NA | NA | NA | NA |
| Fall | Zion Chapel | 1 |  | 5.8 | 4.4 |  | 3.9 | 6.4 | 10.1 |  | 2.1 | 4.3 | 6.9 |  | NA | NA | NA | NA |
| Spring | Burks Mountain | 4 |  | 9.3 | 6.1 |  | 3.5 | 9.3 | 21.4 |  | 3.9 | 6.4 | 12.7 |  | NA | NA | NA | NA |
| Spring | Childs | 3 |  | 7.8 | 3.7 |  | 5.0 | 6.6 | 10.7 |  | 2.5 | 4.0 | 5.8 |  | 0.0 | 0.5 | 0.0 | 0.5 |
| Spring | Clayton Creek | 4 |  | 6.0 | 3.6 |  | 13.4 | 47.7 | 114.0 |  | 4.9 | 8.3 | 11.8 |  | 0.5 | 0.3 | 0.4 | 0.0 |
| Spring | Dunn | 8 |  | 11.0 | 5.0 |  | 3.2 | 4.2 | 7.0 |  | 3.6 | 6.0 | 8.8 |  | 0.2 | 0.4 | 0.2 | 0.4 |
| Spring | Gaston | 3 |  | 5.3 | 2.4 |  | 4.5 | 8.7 | 14.0 |  | 2.8 | 4.3 | 6.9 |  | NA | NA | NA | NA |
| Spring | Grant | 11 |  | 22.0 | 8.5 |  | 1.3 | 2.2 | 4.2 |  | 7.4 | 9.4 | 13.3 |  | 1.0 | 0.2 | 0.0 | 0.2 |
| Spring | Hwy 82 | 4 |  | 4.2 | 1.4 |  | 6.1 | 13.5 | 20.2 |  | 1.2 | 1.9 | 2.5 |  | 0.3 | 0.4 | 0.1 | 0.5 |
| Spring | Kitchens | 2 |  | 4.1 | 2.2 |  | 4.0 | 6.4 | 8.2 |  | 3.4 | 3.9 | 5.2 |  | 0.2 | 0.2 | 0.2 | 0.3 |
| Spring | Mt. Calvary | 6 |  | 9.0 | 4.1 |  | 1.7 | 5.2 | 9.1 |  | 4.5 | 6.6 | 10.5 |  | 0.9 | 0.0 | 0.0 | 0.1 |
| Spring | Parker Hill | 11 |  | 13.7 | 5.2 |  | 4.8 | 5.8 | 8.1 |  | 4.7 | 8.4 | 9.1 |  | NA | NA | NA | NA |
| Spring | Pines Grove | 5 |  | 12.9 | 6.7 |  | 2.8 | 4.1 | 8.5 |  | 5.2 | 6.7 | 8.2 |  | 0.3 | 0.3 | 0.2 | 0.5 |
| Spring | Saluda | 7 |  | 10.4 | 4.1 |  | 3.2 | 4.8 | 6.7 |  | 5.1 | 7.0 | 10.2 |  | 0.5 | 0.1 | 0.5 | 0.2 |
| Spring | Soap Creek | 5 |  | 7.3 | 2.7 |  | 2.8 | 5.0 | 9.5 |  | 4.3 | 5.8 | 7.7 |  | NA | NA | NA | NA |
| Spring | Zion Chapel | 5 |  | 9.9 | 5.3 |  | 3.3 | 4.5 | 13.0 |  | 5.6 | 7.0 | 8.9 |  | 0.1 | 0.1 | 0.3 | 0.3 |
| Summer | Burks Mountain | 3 |  | 7.9 | 3.4 |  | 5.7 | 7.1 | 10.5 |  | 2.4 | 3.6 | 5.5 |  | NA | NA | NA | NA |
| Summer | Childs | 4 |  | 11.9 | 5.3 |  | 4.1 | 5.4 | 7.8 |  | 3.6 | 4.6 | 5.4 |  | 0.5 | 0.0 | 0.0 | 1.0 |
| Summer | Clayton Creek | 5 |  | 6.3 | 2.4 |  | 23.4 | 60.0 | 89.4 |  | 2.8 | 4.1 | 7.4 |  | 0.0 | 0.5 | 0.5 | 0.1 |
| Summer | Gaston | 3 |  | 4.1 | 1.8 |  | 5.1 | 11.1 | 18.4 |  | 1.5 | 2.2 | 3.8 |  | NA | NA | NA | NA |
| Summer | Grant | 4 |  | 5.1 | 2.1 |  | 4.1 | 8.1 | 13.1 |  | 3.8 | 4.6 | 6.2 |  | 0.5 | 0.2 | 0.0 | 0.5 |
| Summer | Hwy 82 | 3 |  | 4.0 | 1.9 |  | 5.9 | 13.1 | 20.1 |  | 2.2 | 3.2 | 4.5 |  | 0.4 | 0.3 | 0.1 | 0.5 |
| Summer | Kitchens | 5 |  | 6.0 | 2.1 |  | 7.6 | 10.8 | 16.1 |  | 2.1 | 2.8 | 4.2 |  | 0.3 | 0.3 | 0.0 | 0.9 |
| Summer | Mt. Calvary | 5 |  | 6.0 | 2.1 |  | 7.0 | 9.4 | 14.2 |  | 2.3 | 4.2 | 6.8 |  | 1.0 | 0.0 | 0.0 | 0.0 |
| Summer | Parker Hill | 5 |  | 4.9 | 0.9 |  | 2.9 | 3.8 | 5.2 |  | 3.8 | 5.2 | 18.5 |  | NA | NA | NA | NA |
| Summer | Pines Grove | 3 |  | 12.7 | 6.0 |  | 2.8 | 4.8 | 6.9 |  | 3.3 | 4.4 | 5.7 |  | 0.5 | 0.1 | 0.3 | 0.9 |
| Summer | Saluda | 6 |  | 7.3 | 3.0 |  | 4.9 | 8.1 | 13.0 |  | 2.6 | 4.0 | 5.6 |  | 0.3 | 0.8 | 0.1 | 0.0 |
| Summer | Soap Creek | 3 |  | 5.0 | 2.6 |  | 4.7 | 8.5 | 14.4 |  | 1.6 | 2.2 | 3.0 |  | NA | NA | NA | NA |
| Summer | Zion Chapel | 2 |  | 6.2 | 4.6 |  | 10.9 | 28.5 | 60.4 |  | 1.5 | 2.7 | 4.5 |  | 0.1 | 0.3 | 0.3 | 0.4 |
| Winter | Burks Mountain | 3 |  | 5.1 | 2.7 |  | 4.9 | 8.3 | 15.9 |  | 1.3 | 2.6 | 5.1 |  | NA | NA | NA | NA |
| Winter | Childs | 3 |  | 9.5 | 8.7 |  | 3.9 | 9.0 | 16.3 |  | 3.0 | 5.6 | 8.6 |  | 0.0 | 1.0 | 0.0 | 0.0 |
| Winter | Clayton Creek | 6 |  | 8.2 | 3.5 |  | 6.8 | 12.6 | 53.6 |  | 2.6 | 4.4 | 8.7 |  | 0.5 | 0.5 | 0.2 | 0.0 |
| Winter | Dunn | 4 |  | 8.0 | 3.6 |  | 3.0 | 5.2 | 8.6 |  | 2.2 | 4.2 | 5.4 |  | 0.4 | 0.6 | 0.1 | 0.0 |
| Winter | Gaston | 1 |  | 1.4 | 0.5 |  | 0.3 | 2.7 | 5.6 |  | 1.8 | 7.0 | 13.5 |  | NA | NA | NA | NA |
| Winter | Grant | 4 |  | 5.5 | 1.9 |  | 3.3 | 6.1 | 10.7 |  | 4.2 | 5.8 | 9.3 |  | 0.6 | 0.1 | 0.2 | 0.0 |
| Winter | Hwy 82 | 2 |  | 3.6 | 2.8 |  | 3.8 | 6.9 | 15.0 |  | 1.1 | 2.3 | 4.8 |  | 0.5 | 0.5 | 0.0 | 0.0 |
| Winter | Joyes | 2 |  | 4.1 | 2.8 |  | 3.7 | 7.0 | 13.6 |  | 2.8 | 4.1 | 4.8 |  | NA | NA | NA | NA |
| Winter | Mt. Calvary | 6 |  | 9.9 | 5.1 |  | 9.8 | 21.0 | 46.5 |  | 3.4 | 4.8 | 6.7 |  | 0.7 | 0.2 | 0.0 | 0.0 |
| Winter | Parker Hill | 8 |  | 14.3 | 5.8 |  | 3.9 | 4.3 | 5.6 |  | 8.7 | 10.3 | 11.4 |  | 0.5 | 0.4 | 0.1 | 0.0 |
| Winter | Pines Grove | 3 |  | 7.6 | 4.7 |  | 5.3 | 9.8 | 19.0 |  | 2.4 | 4.5 | 8.2 |  | 0.6 | 0.3 | 0.3 | 0.0 |
| Winter | Saluda | 12 |  | 18.6 | 7.8 |  | 2.6 | 3.5 | 7.2 |  | 5.3 | 6.5 | 7.1 |  | NA | NA | NA | NA |
| Winter | Soap Creek | 2 |  | 3.7 | 1.8 |  | 5.6 | 10.4 | 24.3 |  | 1.8 | 2.6 | 3.6 |  | 0.4 | 0.3 | 0.5 | 0.1 |
| Winter | Zion Chapel | 1 |  | 6.7 | 3.1 |  | 2.8 | 5.5 | 13.7 |  | 3.4 | 6.3 | 9.4 |  | 0.2 | 0.2 | 0.5 | 0.0 |

^a^White-tailed deer

^b^Small mammals
